# Impact of shear wave dispersion slope analysis for assessing the severity of myocarditis

**DOI:** 10.1038/s41598-022-12935-6

**Published:** 2022-05-24

**Authors:** Naofumi Amioka, Yoichi Takaya, Kazufumi Nakamura, Megumi Kondo, Kaoru Akazawa, Yuko Ohno, Keishi Ichikawa, Rie Nakayama, Yukihiro Saito, Satoshi Akagi, Toru Miyoshi, Masashi Yoshida, Hiroshi Morita, Hiroshi Ito

**Affiliations:** 1 Department of Cardiovascular Medicine, Okayama University Graduate School of Medicine, Dentistry and Pharmaceutical Sciences, 2-5-1, Shikata-cho, Kita-ku, Okayama, 700-8558 Japan; 2 Kawasaki University of Medical Welfare, Okayama, Japan

**Keywords:** Preclinical research, Translational research

## Abstract

This study aimed to elucidate the utility of a novel ultrasound-based technique, shear wave dispersion slope (SWDS) analysis, which estimates tissue viscosity, for evaluating the severity of myocardial inflammation. Experimental autoimmune myocarditis (EAM) at different disease phases [3-week (acute phase): n = 10, 5-week (subacute phase): n = 9, and 7-week (late phase): n = 11] were developed in male Lewis rats. SWDS was measured in the right and the left ventricular free walls (RVFW and LVFW) under a retrograde perfusion condition. Histological myocardial inflammation was evaluated by CD68 staining. The accumulation of CD68-positive cells was severe in the myocardium of the EAM 3-week group. The median (interquartile range) SWDS of RVFW was significantly higher in the EAM 3-week group [9.9 (6.5–11.0) m/s/kHz] than in the control group [5.4 (4.5–6.8) m/s/kHz] (P = 0.034). The median SWDS of LVFW was also significantly higher in the EAM 3-week group [8.1 (6.4–11.0) m/s/kHz] than in the control group [4.4 (4.2–4.8) m/s/kHz] (P = 0.003). SWDS and the percentage of CD68-positive area showed a significant correlation in RVFW (R^2^ = 0.64, P < 0.001) and LVFW (R^2^ = 0.73, P < 0.001). This study showed that SWDS was elevated in ventricular walls with acute inflammation and also significantly correlated with the degree of myocardial inflammation. These results suggest the potential of SWDS in estimating the histological severity of acute myocarditis.

## Introduction

Myocarditis is an inflammatory heart disease that has been reported a high mortality rate^1^.

To improve the prognosis of patients, diagnosing and assessing the severity of myocarditis play important roles in clinical practice, while they still depend on endomyocardial biopsy. However, because endomyocardial biopsy is an invasive procedure, it is accompanied by a risk of complications^2,3^. Although several studies reported the usefulness of cardiac magnetic resonance imaging and ^18^F-fluoro-d-glucose positron emission tomography imaging as non-invasive tools for the assessment of myocarditis, these imaging modalities are difficult to perform in patients with severe disease conditions^4,5^. The diagnosis of the severity of myocarditis at the acute phase remains challenging.

**Figure 1:**
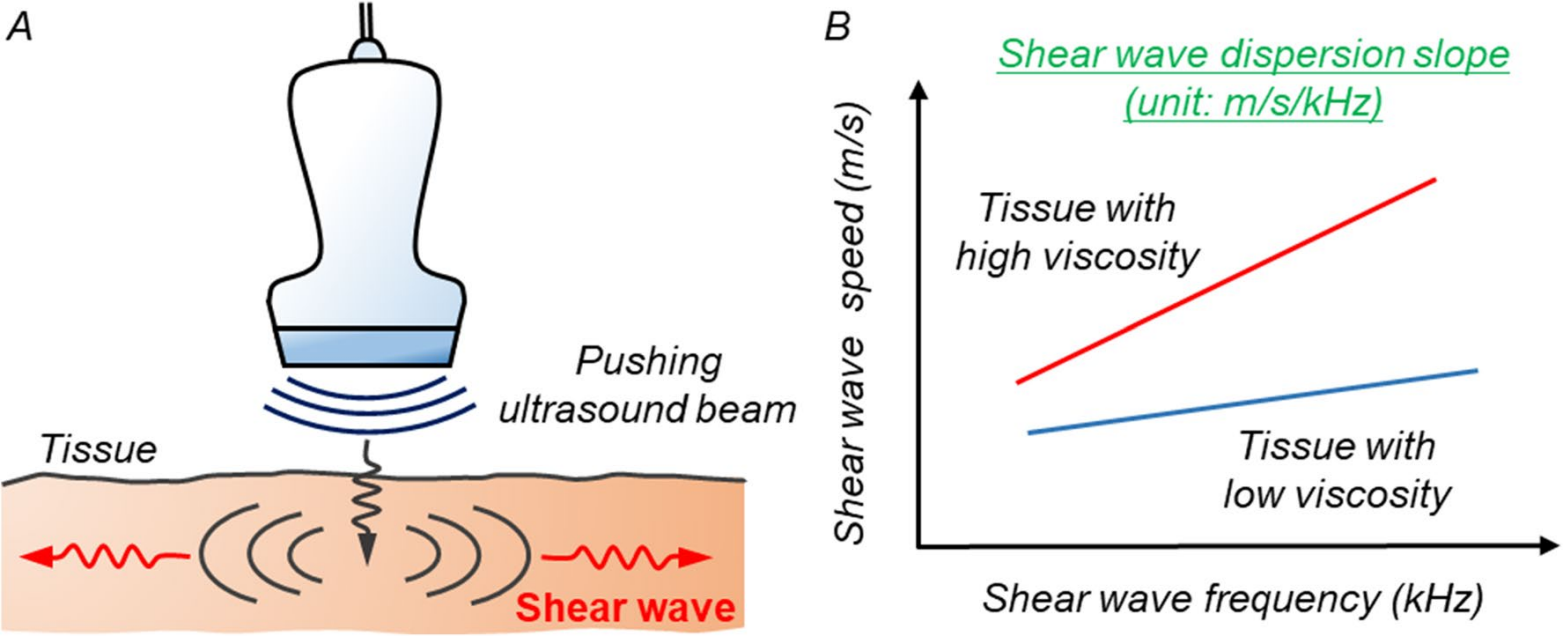
Mechanism of shear wave dispersion slope for assessing tissue viscosity. (**A**) Mechanism of shear wave generation in biological tissue. (**B**) Relationship between shear wave dispersion slope and tissue viscosity.

Shear wave (SW) imaging is a novel ultrasound technology for assessing the characteristics of tissues. SW is generated as a laterally propagating wave by an acoustic radiation force impulse, which is caused by pushing an ultrasound beam (Fig. 1A). SW speed depends on two characteristics of materials, such as elasticity and viscosity. SW elasticity, which is calculated by SW speed, has been reported to reflect tissue stiffness in various organs, including the liver, thyroid, and breast^6–10^. In the field of cardiovascular diseases, Villemain et al. reported that myocardial stiffness during end-diastole calculated by SW speed was significantly higher in patients with hypertrophic cardiomyopathy than in healthy volunteers^11,12^.

Recently, SW dispersion slope (SWDS) has been attracting attention as a method for evaluating tissue viscosity. SW speed depends on the frequency of SW in a viscoelastic tissue^13^. The gradient of SW speed, that is, the slope of SW speed versus SW frequency is changed in response to viscosity. Therefore, SWDS can be used to estimate tissue viscosity (Fig. 1B). Sugimoto et al. reported that SWDS was significantly correlated with the grade of inflammation in hepatic tissue in rat models^14^. Based on these researches, we hypothesized that SWDS can diagnose myocardial inflammation and assess its severity. This study aimed to elucidate the utility of SWDS for evaluating the severity of myocarditis in rats of experimental autoimmune myocarditis (EAM) models with various disease phases.

## Methods

### EAM models and experiment protocol

Forty-two male Lewis rats (Charles River Laboratories, Kanagawa, Japan), aged 7 weeks and weighing 150–200 g, were purchased, and were housed under standard conditions (temperature 23 ± 1 ℃, humidity 50 ± 60%, 12-h light–dark cycle), with food in the form of dry pellets and tap water available ad libitum throughout the study. To induce EAM, we subjected the rats to porcine cardiac myosin (PCM) (M0531, Sigma Aldrich, St. Louis, MO, USA) immunization, as previously described^5^. In brief, PCM was dissolved in phosphate-buffered saline (PBS) at 10 mg/mL and emulsified with an equal volume of complete Freund's adjuvant (CFA) with 1 mg/mL *Mycobacterium tuberculosis* H37RA (BD 231131, Difco Lab., Detroit, MI, USA). EAM in rats was induced by immunization with 0.2 ml of PCM-CFA emulsion (containing 1 mg of PCM) by subcutaneous injection into the rear footpad under inhalation anesthesia using 3% isoflurane, and 7 days later, rats were received second immunization by the same method. In this study, rats were divided into four groups: the control group (n = 10), the EAM 3-week group (n = 11) as an acute phase model, the EAM 5-week group (n = 10) as a subacute phase model, and the EAM 7-week group (n = 11) as a late phase model^5,15^. EAM 3-week group, EAM 5-week group, and EAM 7-week group were received immunization at 11- and 12-week, 9- and 10-week, and 7- and 8-week of age, respectively, as shown on Supplementary Fig. 1. The control group (n = 10) was injected with PBS-CFA emulsion at 7- and 8-week of age. At the end of the study period (14 weeks of age), rats were anesthetized with inhalation of 3% isoflurane and received transthoracic echocardiography. Subsequently, blood was collected via the inferior vena cava, and then heart tissue was harvested to measure SWDS under a retrograde perfusion condition.

All experiments performed in this study were approved by the Okayama University Animal Care and Use Committee (Approval no: OKU-2019618). They were conducted in accordance with the Okayama University Guidelines, which are based on the National Institutes of Health’s Guide for the Care and Use of Laboratory Animal and the ARRIVE (Animal Research: Reporting of In Vivo Experiments) guidelines.

### Transthoracic echocardiography

Transthoracic echocardiography was performed using Aplio ver. 6.0 with a 10-MHz sector probe (Canon Medical Systems, Otawara, Japan). The thickness of the right and left ventricular free walls (RVFW and LVFW) and intraventricular septum, and left ventricular (LV) end-diastolic diameter, LV end-systolic diameter, LV fractional shortening, LV ejection fraction, and right ventricular end-diastolic diameter were measured by M-mode imaging in the parasternal long-axis view.

### Enzyme-linked immunosorbent assay (ELISA)

Serum samples were separated after centrifugation of blood samples at 3,000 rpm for 20 min. Serum troponin I (ab246529, Abcam, Cambridge, UK) and serum interleukin-6 (R6000B, R&D Systems, Minneapolis, MN, USA) levels were measured by ELISA kits.

### Retrograde perfusion system

We performed SW imaging by ex vivo experiment because the heart rate of rats is too rapid to obtain images. Therefore, a retrograde perfusion system was used to maintain the completely relaxed condition of rat's hearts as previously reported^16–18^. In brief, after the sacrifice of rats, hearts were excised and immediately submerged in the Tyrode’s solution (136 mmol/L NaCl, 5.4 mmol/L KCl, 1.8 mmol/L CaCl2, 0.53 mmol/L MgC12, 5.5 mmol/L HEPES, and 1% Glucose, pH 7.4, 37℃) added with 20 mmol/L butanedione monoxime, an inhibitor of actin-myosin interaction, and 10 μmol/L blebbistatin, a specific myosin II inhibitor. The ascending aorta was cannulated with an 18-gauge blunted needle connected to a retrograde perfusion system. The heart was perfused with the Tyrode’s solution added butanedione monoxime and blebbistatin to induce complete relaxation^16^.

### SW imaging

The heart completely relaxed by the retrograde perfusion system was set in a water tank of agar phantom (Model 049A, CIRS, Norfolk, VA, USA). SW imaging was performed using Aplio i900 with an 18-MHz linear probe (Canon Medical Systems) by two cardiologists who did not know the background of samples. The B-mode image was obtained in the parasternal long-axis view. A rectangular region of interest (ROI) was placed on RVFW and LVFW. SW in the tissue was generated by pushing pulse of 5–18 MHz^17^. After confirming proper SW propagation in a “wave front” style display, SW speed was obtained based on the tissue Doppler technique. For the evaluation of SWDS, the displacement of the tissue caused by the SW was obtained by a technique based on tissue Doppler imaging. The displacement of the tissue at each beam position was transformed from time domain into frequency domain by Fourier transformation to estimate the phase change of SW in the lateral direction at several frequencies. SW speed c(ω) at each frequency was calculated using the phase-gradient method: c(ω) = ω ΔL/Δφ, where ΔL and Δφ are the distance and the phase difference measured between two detection points along the SW propagation path. SWDS, which is the gradient of SW speed, was calculated based on the distribution of SW speed versus SW frequency^19^. Also, SW elasticity was measured using the equation: 3ρc^2^, where ρ is the density of the tissue. A circular ROI of 1-mm in diameter was placed at the mid-level of RVFW and LVFW, and SWDS and SW elasticity were measured automatically. Each measurement was repeated five times, and the average value was compared between each group. The setting of generation and analysis of SW was kept for all cardiac wall segments during the experiment.

### Histological assessment

After SW imaging, heart weight was measured to calculate relative heart weight against body weight. The heart was sectioned transversely at the mid-papillary level, and then fixed with 10% formalin, embedded in paraffin, and cut into 5-μm sections. Sections were stained with hematoxylin–eosin for evaluating infiltrating inflammatory cells, and with picrosirius red for evaluating fibrosis. Macrophages, which accounted for the majority of infiltrating inflammatory cells in EAM model, were identified by mouse anti-rat CD68 monoclonal antibody (ab31630, Abcam) staining. The CD68-positive area was quantitatively calculated using ImageJ software (version 1.52v, National Institutes of Health, Bethesda, MD, USA) by setting an intensity threshold that matched the visually identified staining areas as previously reported^5^. Similar to SW imaging, the percentage of CD68-positive area in a circular ROI of 1-mm in diameter was measured at five locations, and the average value was calculated. The percentage of CD68-positive area was compared between each group. The relationship between SWDS and the percentage of CD68-positive area was analyzed to examine whether SWDS reflects pathologically evaluated myocardial inflammation.

### Statistical analysis

Samples obtained from all rats except those who died during the study period were included in each analysis. Statistical analysis was carried out with R (The R Foundation for Statistical Computing, Vienna, Austria)^20^ or SigmaPlot version 14.5 (Systat Software Inc., San Jose, CA, USA). The Shapiro–Wilk test was used to check the normality of data. Data are expressed as means ± standard deviation for normally distributed continuous variables and the median (interquartile range) for non-normally distributed continuous variables. We used one-way analysis of variance to compare normally distributed continuous variables. Kruskal–Wallis analysis of median test was used for comparing non-normally distributed continuous variables. Bonferroni correction was applied for post hoc comparisons between two groups. Pearson correlation coefficient was analyzed to evaluate the relationship of SWDS with the percentage of CD68-positive area, log-transformed serum troponin I level, or serum interleukin-6 level. Multivariate linear regression analysis was used to evaluate the impact of SWDS as a predictor for CD68-positive area in ventricular walls. Differences with P < 0.05 were considered significant.

## Results

### Clinical course of animals

One rat in the EAM 3-week group and one rat in the EAM 5-week group died during the acute phase of EAM. One rat in the control group died from infection secondary to arthritis caused by the injection of complete Freund’s adjuvant. Finally, a total of 39 rats were used for analyses in this study [the control group (n = 9), the EAM 3-week group (n = 10), the EAM 5-week group (n = 9), and the EAM 7-week group (n = 11)].

### Basic data of each group

Representative images of the heart in each group, excised at 14 weeks age, were shown in Supplementary Fig. 2. The heart in the EAM 3-week group showed a severe edematous appearance. Heart weight and relative heart weight in the EAM 3-week group were significantly heavier than those in the control group and the EAM 7-week group (Supplementary Table 1). The thickness of RVFW, LVFW, and intraventricular septum was increased in the EAM 3-week and the EAM 5-week groups, compared to the control group. In addition, all those parameters showed the highest in the EAM 3-week group. In the EAM 5-week and EAM 7-week groups, LV end-diastolic diameter was significantly larger than that in the EAM 3-week group. LV end-systolic diameter was increased in all EAM groups, compared to the control group. In addition, LV end-systolic diameter in the EAM 5-week and EAM 7-week groups was significantly larger than that of the EAM 3-week group. LV fractional shortening and LV ejection fraction were significantly decreased in all EAM groups, compared to the control group. Serum levels of troponin I and interleukin-6 of the EAM 3-week group were 451.8 pg/ml and 122.2 ± 30.5 pg/ml, respectively, and those parameters were significantly higher in the EAM 3-week group than in other groups. The details of basic data of each group are shown in Supplementary Table 1.

### Histological evaluation

**Figure 2:**
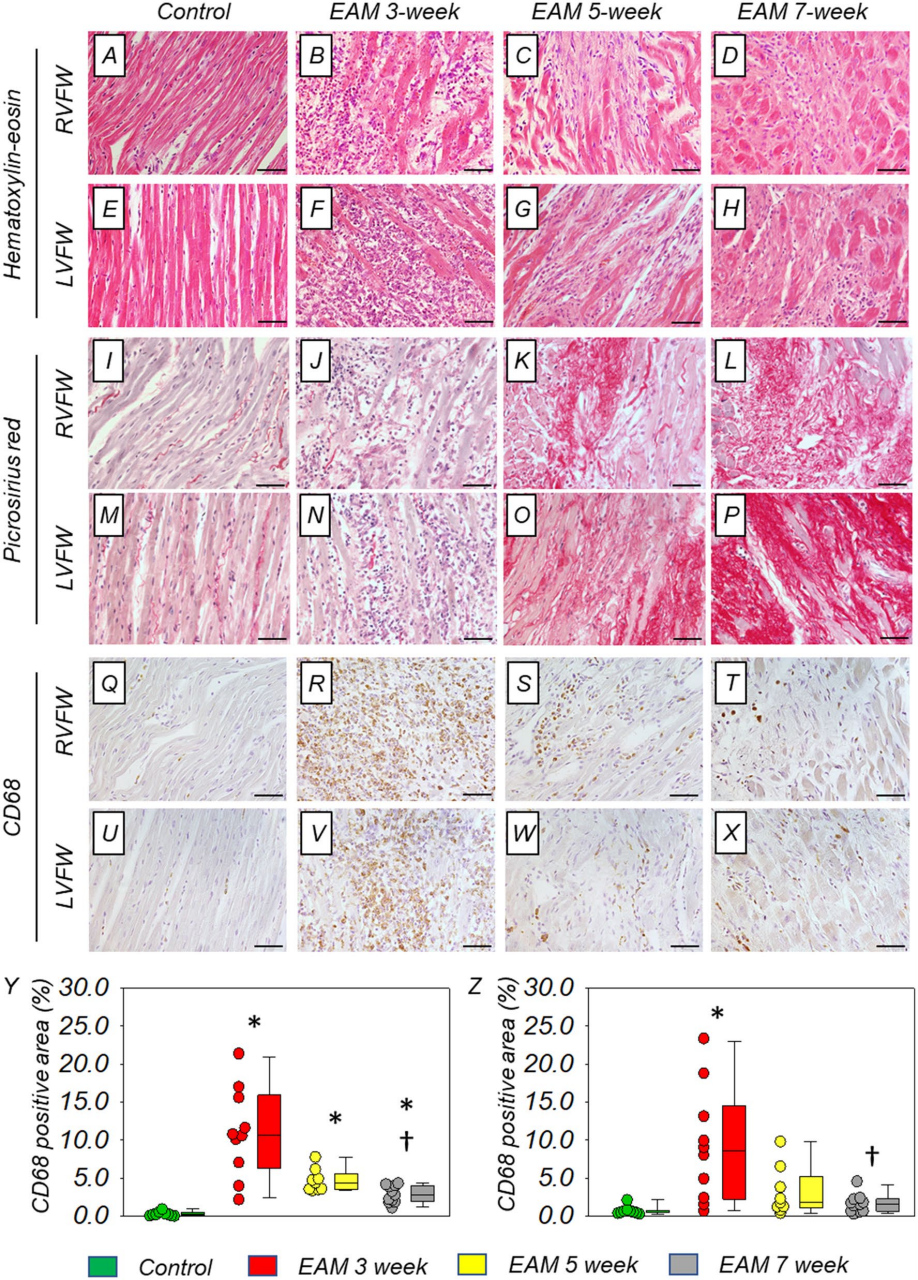
Histological evaluation of myocarditis. (**A–H**) Hematoxylin–eosin staining in both ventricular free walls. (**I–P**) Fibrosis of both ventricular free walls stained with picrosirius red (shown as vivid red color). (**Q–X**) Infiltration of macrophages of both ventricular free walls stained with anti-rat CD68 antibody (shown as brown color). Scale bars = 50 μm. Comparison of the percentage of CD68-positive area of RVFW (**Y**) and LVFW (**Z**) between the groups [control group (n = 9), the EAM 3-week group (n = 10), the EAM 5-week group (n = 9), and the EAM 7-week group (n = 11)]. The CD68-positive area was quantitatively calculated using ImageJ software as described in “Methods” section. Box plots show the median, interquartile range, and minimum/maximum data of the samples. *P < 0.05 vs the control group. ^†^P < 0.05 vs EAM 3-week group, in the EAM 5-week group or EAM 7-week group. *EAM* experimental autoimmune myocarditis, *LVFW* left ventricular free wall, *RVFW* right ventricular free wall.

Histological findings of the myocardium were shown in Fig. 2. In RVFW and LVFW, hematoxylin–eosin staining showed more severe infiltration of inflammatory cells accompanied with extracellular edema in the EAM 3-week group, compared to the EAM 5-week group and the EAM 7-week group (Fig. 2A–H). Fibrosis identified by picrosirius red staining was trivial in the control group and the EAM 3-week group, while that was moderate in the EAM 5-week group and severe in the EAM 7-week group (Fig. 2I–P). The staining by anti-rat CD68 antibody showed severe infiltration of macrophages in both ventricular free walls of the EAM 3-week group. On the other hand, the infiltration of macrophages was reduced in the EAM 5-week group and the EAM 7-week group (Fig. 2Q–X). The percentage of CD68-positive area of RVFW was significantly increased in EAM groups, compared to the control group [0.2 (0.1–0.4) %] (all P < 0.001). There was no statistically significant difference between the EAM 3-week group [10.7 (6.3–16.0) %] and the EAM 5-week group [4.4 (3.5–5.5) %], while the EAM 3-week group showed higher percentage of CD68-positive area in RVFW, compared to the EAM 7-week group [2.8 (1.9–4.0) %] (P = 0.005) (Fig. 2Y). In LVFW, the percentage of CD68-positive area was significantly increased only in the EAM 3-week group [8.5 (2.2–14.5) %], not in the EAM 5-group [1.8 (1.0–5.2) %] or the EAM 7-week group [1.6 (0.7–2.2) %], compared to the control group [0.5 (0.4–0.7) %] (P < 0.001). The EAM 3-week group also showed higher percentage of CD68-positive area than the EAM 7-week group in LVFW (P = 0.017) (Fig. 2Z).

### SW imaging and myocardial inflammation at various phases of EAM

**Figure 3:**
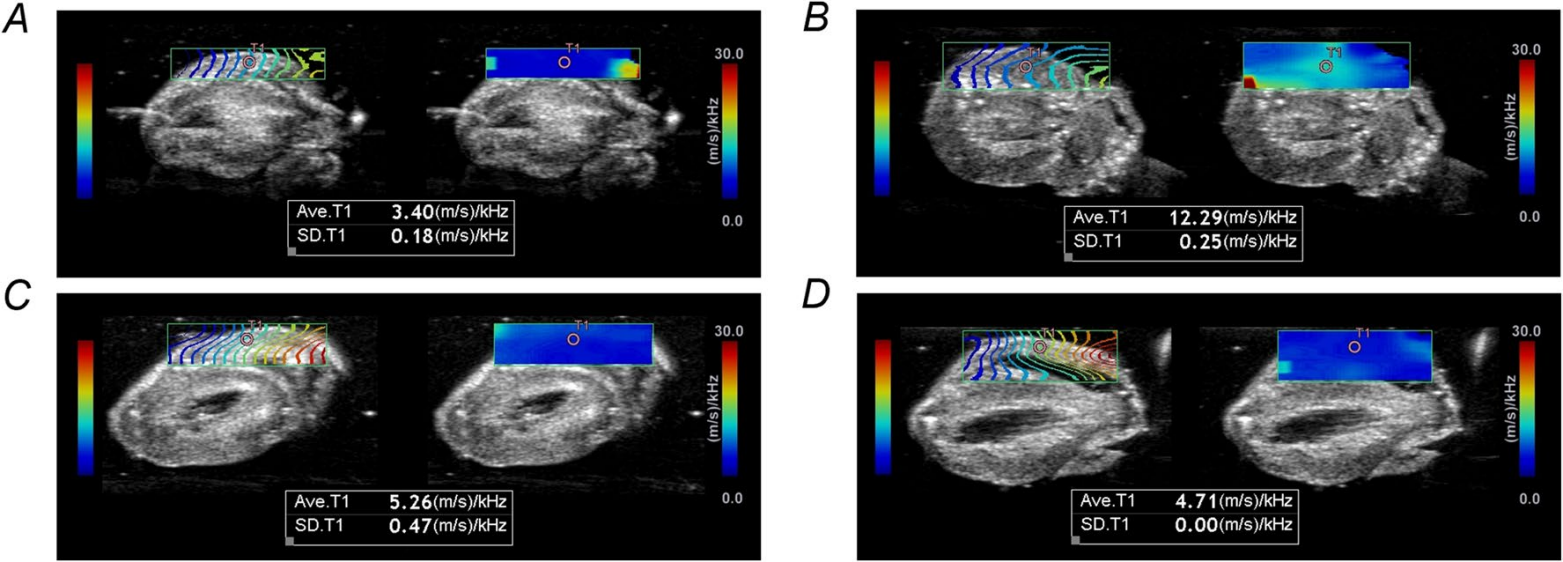
SWDS images. SW propagation image (left side) and SWDS image (right side) of RVFW in the control group (**A**), the EAM 3-week group (**B**), the EAM 5-week group (**C**), and the EAM 7-week group (**D**). The value of SWDS in a circular ROI of 1-mm in diameter on the myocardium was measured. *EAM* experimental autoimmune myocarditis, *ROI* region of interest, *RVFW* right ventricular free wall, *SW* shear wave, *SWDS* shear wave dispersion slope.

Representative SW propagation images and SWDS images in each group were shown in Fig. 3 (RVFW) and Supplementary Fig. 3 (LVFW). The value of SWDS was visually shown by using color distribution set in the range of 0–30 m/s/kHz. SWDS images in the control group, the EAM 5-week group, and the EAM 7-week group were blue or light blue color, which meant a low value of SWDS. On the other hand, the EAM 3-week group showed light green color, which meant a relative high value of SWDS in both of RVFW and LVFW.

**Figure 4:**
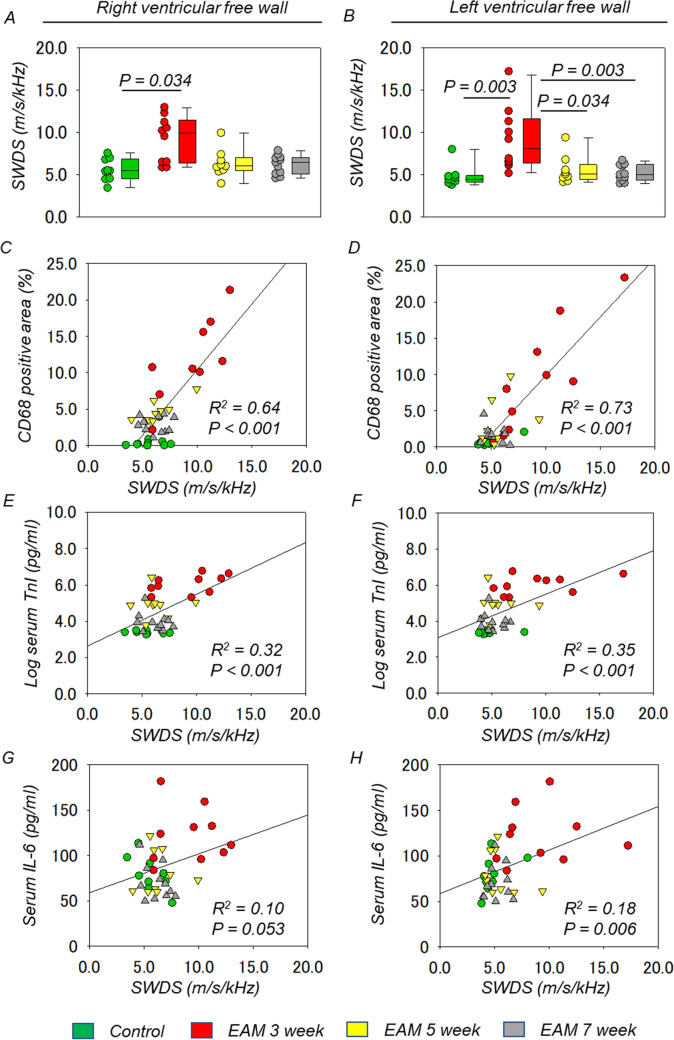
SWDS and the severity of myocardial inflammation. Comparison of SWDS of RVFW (**A**) and LVFW (**B**) between the groups. Box plots show the median, interquartile range, and minimum/maximum data of the samples [control group (n = 9), the EAM 3-week group (n = 10), the EAM 5-week group (n = 9), and EAM 7-week group (n = 11)]. Relationship between SWDS and the percentage of CD68-positive area in RVFW (**C**) and LVFW (**D**) [the control group (n = 9), the EAM 3-week group (n = 10), the EAM 5-week group (n = 9), and the EAM 7-week group (n = 11)]. Relationship between SWDS and serum troponin I level in RVFW (**E**) and LVFW (**F**). Relationship between SWDS and serum interleukin-6 level in RVFW (**G**) and LVFW (**H**). *EAM* experimental autoimmune myocarditis, *LVFW* left ventricular free wall, *R* pearson correlation coefficient, *RVFW* right ventricular free wall, *SWDS* shear wave dispersion slope.

Comparisons of SWDS of RVFW and LVFW between the groups were shown in Fig. 4A,B. SWDS of RVFW was significantly higher in the EAM 3-week group [9.9 (6.5–11.0) m/s/kHz], compared to the control group [5.4 (4.5–6.8) m/s/kHz] (P = 0.034). In addition, SWDS of RVFW was 6.0 (5.5–6.7) m/s/kHz in the EAM 5-week group and 6.4 (5.2–7.0) m/s/kHz in the EAM 7-week group. There was no other significant difference in SWDS of RVFW between each group. SWDS of LVFW was significantly higher in the EAM 3-week group [8.1 (6.4–11.0) m/s/kHz] than in the control group [4.4 (4.2–4.8) m/s/kHz] (P = 0.003), the EAM 5-week group [5.0 (4.6–5.6) m/s/kHz] (P = 0.034), and the EAM 7-week group [5.0 (4.5–6.1) m/s/kHz] (P = 0.003). There was no other statistically significant difference in SWDS of LVFW between each group.

Figure 4C,D showed correlation diagrams between SWDS and the percentage of CD68-positive area in both ventricular free walls. These two factors showed a significant positive correlation in RVFW (R^2^ = 0.64, P < 0.001) and LVFW (R^2^ = 0.73, P < 0.001). SWDS was also correlated with log-transformed serum troponin I level in RVFW (R^2^ = 0.32, P < 0.001) and LVFW (R^2^ = 0.35, P < 0.001) (Fig. 4E,F) and serum interleukin-6 level in LVFW (R^2^ = 0.18, P = 0.006) (Fig. 4G,H).

Because changes in ventricular geometry can affect SWDS, multiple linear regression analysis with adjustment for ventricular wall thickness and end-diastolic diameter was performed to determine the impact of SWDS a as predictive factor for CD68-positive area in RVFW and LVFW. As a result, after adjustment of those geometric factors, SWDS was still a significant predictor of CD68-positive area in RVFW [standardized regression coefficient (β) = 0.59, P < 0.001] (Supplementary Table 2) and in LVFW (β = 0.92, P < 0.001) (Supplementary Table 3).

Comparisons of SW elasticity of RVFW and LVFW between the groups were shown in Supplementary Fig. 4. SW elasticity tended to be higher in the EAM 3-week group, but there was no significant difference in SW elasticity between the control group and the EAM 3-week group in both RVFW [5.3 (3.4–5.8) kPa vs 5.8 (3.5–11.2)] and LVFW [6.6 (5.3–8.6) kPa vs 8.1 (6.5–11.2) kPa]. The only comparison that showed a significant difference in SW elasticity was the EAM 3-week group versus the EAM 5-week group [5.3 (4.1–6.7) kPa] in LVFW (P = 0.048).

## Discussion

The main results of this study were as follows. First, SWDS of ventricular free wall was significantly increased only in the EAM 3-week group (acute-phase model), compared to the control group. Second, SWDS showed a significantly positive correlation with the percentage of CD68-positive area in RVFW and LVFW. To the best of our knowledge, this is the first study to demonstrate the utility of SWDS analysis for evaluating the severity of myocardial inflammation.

### SW imaging and viscosity in myocardial inflammation

SW technology, which uses acoustic radiation force impulse, is based on the theory that SW speed depends on the elasticity and viscosity of tissue according to Kelvin-Voigt viscoelastic models. By using this model, SW speed Cs at SW frequency ω is calculated as shown below:

$$\text{Cs(}{\omega}\text{)}=\sqrt{\frac{\text{2 (}{\mu}^{2}{+}{\omega}^{2}{\cdot}{\eta}^{2}\text{)}}{{\rho (\mu+}\sqrt{{\mu}^{2}{+}{\omega}^{2}{\cdot}{\eta}^{2}}\text{)}}}$$

where ρ, μ, and η are the density, elasticity, and viscosity of the medium, respectively^21,22^. Generally, viscosity does not change significantly in viscoelastic tissues, therefore it is possible to estimate the change of elasticity by ignoring viscosity (η = 0). However, the viscosity cannot be ignored especially in inflammatory diseases including myocarditis, in which the composition of tissues changes rapidly and dramatically. Sugimoto et al. reported that SWDS was transiently increasing during the acute period of hepatitis in a rodent model, and concluded that the increase in SWDS reflected the elevation of viscosity due to edematous change of hepatic tissues associated with inflammation and necrosis^14^. In our study of myocarditis model, SWDS of myocardial tissue was increased only in the acute phase of inflammation, which is consistent with the result of the acute hepatitis model. Previous studies demonstrated that T2 value of cardiac magnetic resonance imaging was correlated with markers of cardiac injury in patients with myocarditis, by reflecting the degree of acute tissue necrosis and edema^23,24^. The hearts of rats with the acute phase of EAM in our study also showed severe infiltration of macrophages accompanied with extracellular edema. Although we could not quantify the edema of myocardial tissue in this study, considering the results of these previous studies, it was suggested that the transient increase in SWDS of the heart in the acute phase indicated myocardial inflammation by reflecting edematous change of tissue.

SW elasticity has been reported to predict the degree of fibrosis in liver tissue^7,25^. However, in our study, SW elasticity of the ventricular wall tended to increase in the acute phase of EAM, when myocardial tissue had not developed significant fibrosis. Because the previous reports showed that SW elasticity in the liver is correlated with tissue stiffness^26,27^, the increase of SW elasticity in ventricular walls might be affected by the elevation of tissue pressure due to myocardial necrosis and edema developed in the acute phase of EAM. In addition, SW elasticity of ventricular walls was not statistically increased in the late phase of EAM. The results of our study could not support the potential of SW elasticity as an alternative to late gadolinium enhancement of cardiac magnetic resonance imaging for evaluating myocardial fibrosis.

### Translational potential of SWDS

In this study, SWDS of ventricular walls was significantly higher in the acute phase of EAM, not in the subacute or late phase of EAM, compared to the control group. This result suggests that SWDS can be a useful modality for detecting the acute phase of myocarditis non-invasively in patients with myocarditis. Because early diagnosis of myocarditis leads to an improvement in prognosis by appropriate treatments, the assessment of SWDS may be valuable in clinical practice. In addition, SWDS analysis have also a potential to be a useful tool for diagnosing other inflammatory diseases, such as cardiac sarcoidosis. Because SWDS image, as shown in Fig. 3, enables visualization of SWDS in myocardial tissue in real time, even local inflammation of myocardium may be able to be determined. This would contribute to reducing the sampling error of endomyocardial biopsy. The clinical usefulness of SWDS analysis has been reported in liver diseases^28,29^. In the field of cardiovascular disease, the effectiveness of measuring SW speed for evaluating myocardial tissue stiffness during end-diastole was also reported in patients with hypertrophic cardiomyopathy^11,12^. Therefore, clinical application of SWDS analysis, which is the same technology, seems to be feasible. However, there are several issues to be resolved. Cardiac geometry is reported to be affected when measuring SW propagation^30^. Furthermore, although patients with fulminant myocarditis often have sinus tachycardia or tachyarrhythmias, the appropriate heart rate for assessing SW imaging is unclear. In addition, a frequency analysis of SW is known to be difficult due to the limited signal-to-noise ratio^31^. Further studies are needed to establish the clinical application of SWDS for myocardial diseases including myocarditis.

### Study limitations

First, this study did not perform SWDS analysis in vivo because the heart rate of rats is too rapid (approximately, 350–400 beats per minutes) to obtain SW imaging. We analyzed SWDS under a retrograde perfusion system to make the heart fully relaxed for mimicking the end-diastolic state, however the measurement under the non-beating condition was the major limitation of this study. Second, SW interacts with the boundaries of tissue, resulting in reflections and mode conversions, guiding SW along the cardiac wall, thus tissue geometric factors including the wall thickness or curvature can affect the value of SWDS. We could not fully include ventricular geometric factors, for example, wall curvature, in multivariate linear regression analysis for evaluating predicting factors of CD68-positive area in ventricular walls. Therefore, there is a possibility that the impact of SWDS as a predictor of myocardial inflammation was overestimated in this study. Third, we measured SWDS of the heart in the long-axis view, because the heart was too small to obtain sufficient area for placing ROI in the short-axis view. On the other hand, histological evaluation was performed in the short-axis view. The measurement sites of SWDS analysis and CD68-positive area might be mismatched.

## Conclusions

SWDS was elevated in the ventricular wall accompanied with acute myocardial inflammation. SWDS was significantly correlated with the degree of myocardial inflammation. SWDS analysis can be a novel method for evaluating the severity of myocarditis by reflecting the viscosity of myocardial tissue.

## Supplementary Information

Supplementary Information.

## Data Availability

The datasets used and/or analyzed during the current study available from the corresponding author on reasonable request.
