# Supplementary material for: Impact of shear wave dispersion slope analysis for assessing the severity of myocarditis
Source: Sci Rep. 2022 May 24;12:8776. doi: 10.1038/s41598-022-12935-6 (PMC9130270; doi:10.1038/s41598-022-12935-6)
Supplement: Supplementary file 1 — Supplementary Information. [file 41598_2022_12935_MOESM1_ESM.docx]

**Supplemental Data**

**Supplementary Table 1. The** **value of basic data of each group.**

|  | Control  (n = 9) | EAM 3-week  (n = 10) | EAM 5-week  (n = 9) | EAM 7-week  (n = 11) |
| --- | --- | --- | --- | --- |
| Heart weight, mg | 1276 ± 112 | 2054 ± 190* | 1919 ± 181* | 1618 ± 174*† |
| Relative heart weight, mg/g | 4.1 ± 0.4 | 6.7 ± 0.7* | 6.2 ± 0.7* | 5.0 ± 0.4*† |
| RVFW thickness, mm | 0.7 ± 0.1 | 1.2 ± 0.2* | 0.9 ± 0.1*† | 0.8 ± 0.1† |
| RV end-diastolic diameter, mm | 6.7 ± 1.6 | 7.4 ± 2.6 | 5.6 ± 0.8 | 5.4 ± 1.8 |
| Interventricular septum thickness, mm | 1.2 ± 0.1 | 1.9 ± 0.2* | 1.7 ± 0.2*† | 1.4 ± 0.1† |
| LVFW thickness, mm | 1.3 ± 0.2 | 2.0 ± 0.2* | 1.7 ± 0.2*† | 1.4 ± 0.1† |
| LV end-diastolic diameter, mm | 7.2 ± 0.4 | 6.5 ± 1.1 | 7.7 ± 0.9† | 7.9 ± 0.4*† |
| LV end-systolic diameter, mm | 3.9 ± 0.4 | 4.7 ± 1.0* | 5.7 ± 1.0*† | 5.9 ± 0.5*† |
| LV fractional shortening, % | 46 ± 4 | 28 ± 7* | 26 ± 7* | 26 ± 5* |
| LV ejection fraction, % | 84 ± 4 | 62 ± 12* | 58 ± 12* | 59 ± 8* |
| Serum troponin I, pg/ml | 29.7 (28.0-33.1) | 451.8 (291.5-571.7)* | 138.2 (132.2-150.9)*† | 51.32 (39.3-59.0)*† |
| Serum interleukin-6, pg/ml | 76.1 ± 21.4 | 122.2 ± 30.5* | 81.3 ± 24.1† | 70.5 ± 19.8† |

Values are means ± standard deviation or median (interquartile range).

EAM, experimental autoimmune myocarditis; LV, left ventricular; LVFW, left ventricular free wall; RV, right ventricular; RVFW, right ventricular free wall.

* P < 0.05 vs control group. † P < 0.05 vs EAM 3-week group in EAM 5-week or EAM 7-week groups.

**Supplementary Table 2. Impact of SWDS as a predictor of CD68-positive area in RVFW evaluated by univariate and multivariate regression analysis.**

|  | B | SE_B_ | β | 95% CI of B | P-value |
| --- | --- | --- | --- | --- | --- |
|  |  |  |  |  |  |
|  |  |  |  |  |  |
| Model 1 | 1.80 | 0.22 | 0.81 | 1.36 – 2.24 | < 0.001 |
| Model 2 | 1.32 | 0.19 | 0.59 | 0.94 – 1.69 | < 0.001 |
| Model 3 | 1.31 | 0.18 | 0.59 | 0.94 – 1.68 | < 0.001 |

Model 1: unadjusted.

Model 2: adjusted for RVFW thickness.

Model 3: adjusted for RVFW thickness and RV end-diastolic diameter.

B: regression coefficient; β: standardized coefficient of B; CI: confidence interval; RV: right ventricular; RVFW: right ventricular free wall.

**Supplementary Table 3. Impact of SWDS as a predictor of CD68-positive area in LVFW evaluated by univariate and multivariate regression analysis.**

|  | B | SE_B_ | β | 95% CI of B | P-value |
| --- | --- | --- | --- | --- | --- |
|  |  |  |  |  |  |
|  |  |  |  |  |  |
| Model 1 | 1.62 | 0.16 | 0.86 | 1.23 – 1.95 | < 0.001 |
| Model 2 | 1.55 | 0.20 | 0.82 | 1.16 – 1.95 | < 0.001 |
| Model 3 | 1.73 | 0.22 | 0.92 | 1.29 – 2.17 | < 0.001 |

Model 1: unadjusted.

Model 2: adjusted for LVFW thickness.

Model 3: adjusted for LVFW thickness and LV end-diastolic diameter.

B: regression coefficient; β: standardized coefficient of B; CI: confidence interval; LV: left ventricular; RVFW: left ventricular free wall.

**
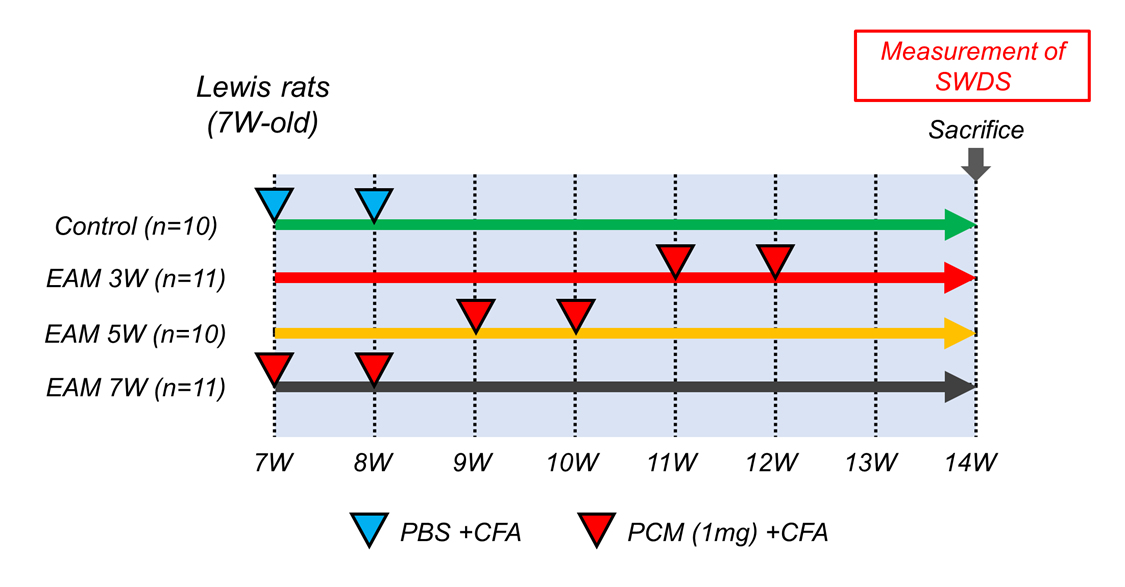
**

**Supplementary Figure 1. EAM models protocol.**

CFA, complete Freund’s adjuvant; EAM, experimental autoimmune myocarditis; PBS, phosphate buffered saline; PCM, porcine cardiac myosin; SWDS, shear wave dispersion slope, W, week.


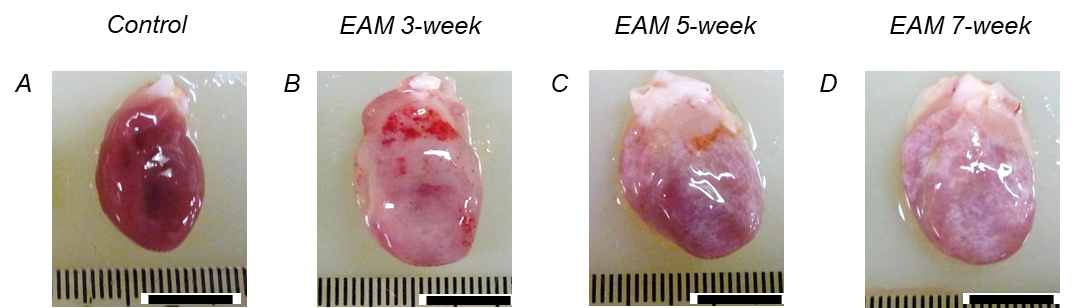


**Supplementary Figure 2. Representative images of the heart in each group.**

Hearts from the control group (A), the EAM 3-week group (B), the EAM 5-week group (C), and the EAM 7-week group (D), respectively. Scale bars = 1 cm.

EAM, experimental autoimmune myocarditis.


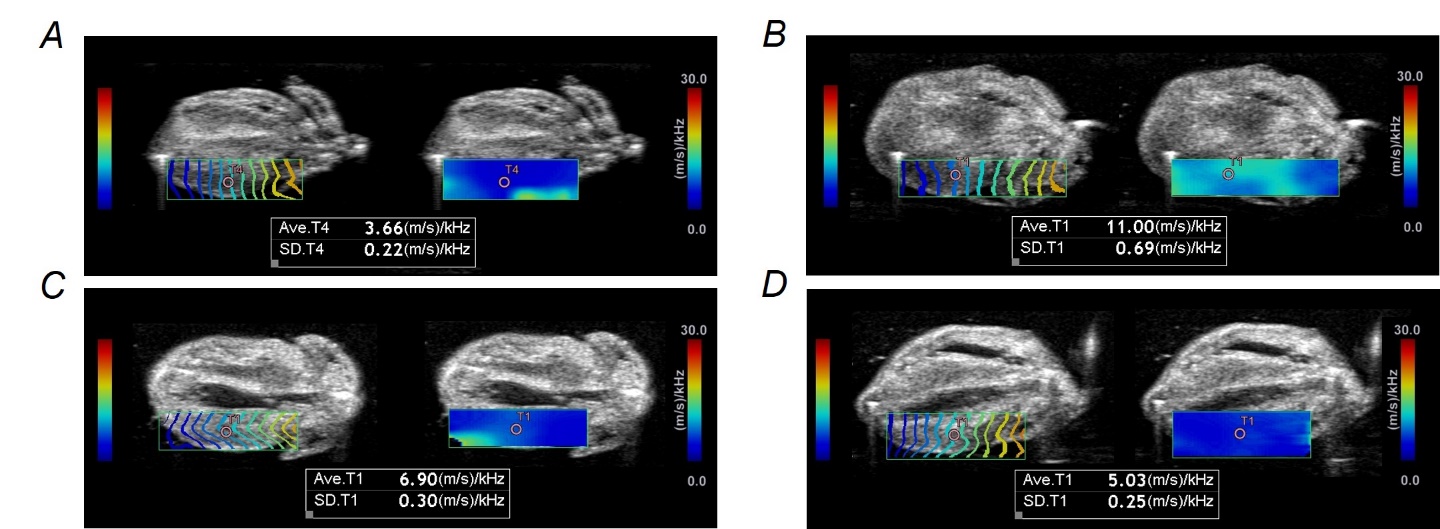


**Supplementary Figure 3. SWDS images of LVFW.**

SW propagation image (left side) and SWDS image (right side) of LVFW in the control group (A), the EAM 3-week group (B), the EAM 5-week group (C), and the EAM 7-week group (D). The value of SWDS in a circular ROI of 1-mm in diameter on the myocardium was measured.

EAM, experimental autoimmune myocarditis; LVFW, left ventricular free wall; ROI, region of interest; SW, shear wave; SWDS, shear wave dispersion slope.


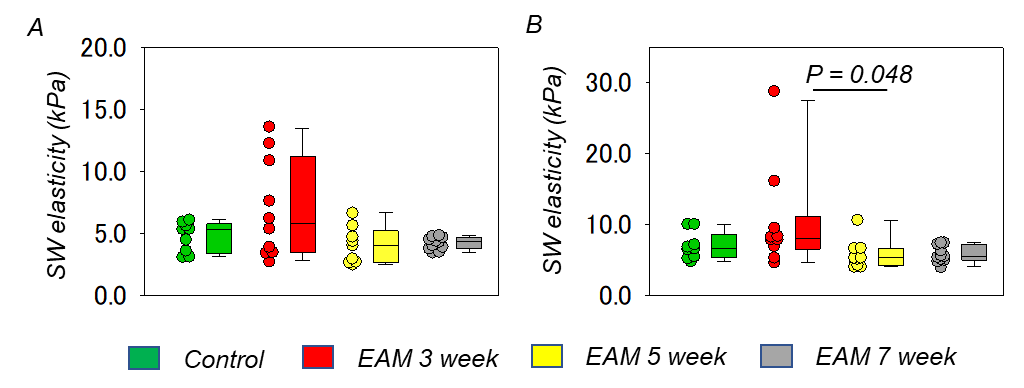


**Supplementary Figure 4. SW elasticity of ventricular walls in different disease phases of EAM.**

Comparison of SW elasticity of RVFW (A) and LVFW (B) between the groups. Box plots show the median, interquartile range, and minimum/maximum data of the samples [the control group (n = 9), the EAM 3-week group (n = 10), the EAM 5-week group (n = 9), and the EAM 7-week group (n = 11)].

EAM, experimental autoimmune myocarditis; LVFW, left ventricular free wall; RVFW, right ventricular free wall; SW, shear wave.
